# Runx-dependent expression of PKC is critical for cell survival in the sea urchin embryo

**DOI:** 10.1186/1741-7007-3-18

**Published:** 2005-08-02

**Authors:** Carrie Dickey-Sims, Anthony J Robertson, Dawn E Rupp, John J McCarthy, James A Coffman

**Affiliations:** 1 Stowers Institute for Medical Research, 1000 E. 50^th ^Street, Kansas City, Missouri, 64110; 2 Department of Physiology, University of Kentucky College of Medicine, Lexington, Kentucky, 40506, USA

## Abstract

**Background:**

Runx transcription factors play critical roles in the developmental control of cell fate and contribute variously as oncoproteins and tumor suppressors to leukemia and other cancers. To discover fundamental Runx functions in the cell biology of animal development, we have employed morpholino antisense-mediated knockdown of the sea urchin Runx protein SpRunt-1. Previously we showed that embryos depleted of SpRunt-1 arrest development at early gastrula stage and underexpress the conventional protein kinase C SpPKC1.

**Results:**

We report here that SpRunt-1 deficiency leads to ectopic cell proliferation and extensive apoptosis. Suppression of the apoptosis by pharmacological inhibition of caspase-3 prevents the ectopic proliferation and rescues gastrulation, indicating that many of the overt defects obtained by knockdown of SpRunt-1 are secondary to the apoptosis. Inhibition or knockdown of SpPKC1 also causes apoptosis, while cell survival is rescued in SpRunt-1 morphant embryos coinjected with *SpPKC1 *mRNA, suggesting that the apoptosis associated with SpRunt-1 deficiency is caused by the deficit in *SpPKC1 *expression. Chromatin immunoprecipitation indicates that SpRunt-1 interacts physically with *SpPKC1 *in vivo, and cis-regulatory analysis shows that this interaction activates *SpPKC1 *transcription.

**Conclusions:**

Our results show that Runx-dependent activation of *SpPKC1 *is essential for maintaining protein kinase C activity at levels conducive to cell survival during embryogenesis.

## Background

The Runt domain (Runx) is a highly conserved 128 amino acid sequence that defines a metazoan family of sequence-specific DNA binding proteins important for the developmental control of cell fate [1]. Vertebrates have three *Runx *genes that are essential for the development of specific tissues (blood, bone, and proprioceptive neurons of the dorsal root ganglia, respectively, for *Runx1*, *Runx2*, and *Runx3*), each of which is associated with leukemogenic or carcinogenic mutations [1-4]. Some of these mutations cause loss of Runx function while others are gain-of-function; hence, mammalian *Runx *genes display attributes of both tumor suppressors and proto-oncogenes [4]. Runx proteins have been shown to regulate cell cycle transit via their function as context-dependent transcriptional regulators. For example, Runx1 stimulates the G1 to S phase transition in cultured mammalian cells, which may in part reflect its transcriptional activation of cyclin D3 [5-7]. Runx1 is also an transactivator of the p14^ARF ^tumor suppressor [8], while both Runx1 and Runx2 are transcriptional repressors of the p21^Cip1/Waf1 ^cyclin-dependent kinase inhibitor [9,10]. These findings indicate that Runx is a critical node in the gene regulatory network that controls cell proliferation and/or survival during animal development. Since many of the core linkages in this network are likely to be conserved throughout animal evolution, identification and cis-regulatory analysis of relevant Runx target genes in experimentally tractable animal models should help illuminate how *Runx *genes contribute to human development and health.

The sea urchin embryo is optically accessible and highly amenable to experimental manipulations such as gene transfer and morpholino antisense mediated knockdown, making it a useful system for discovering fundamental roles played by Runx proteins in the cell biology of animal development. Whole genome sequence data [11] as well as genomic Southern blot analysis [12] suggest that, contrary to previous reports [13-15], the sea urchin *Strongylocentrotus purpuratus *has two *Runx *genes (A. Poustka, personal communication; AJR and JAC, unpublished results). Only one of these (*SpRunt-1*, previously referred to as *SpRunt*) is expressed in the embryo [12,14]. *SpRunt-1 *is required for embryogenesis beyond blastula stage [16] and contributes to transcriptional activation of the *CyIIIa actin *gene, a marker of aboral ectoderm differentiation [12]. *SpRunt-1 *mRNA is expressed globally in the early embryo, whereas in the larva it accumulates predominantly in oral ectoderm and endomesoderm, tissues associated with continued growth and cell proliferation [14]. Embryos depleted of SpRunt-1 by morpholino antisense oligonucleotides (MASOs) that sequence-specifically block either translation or pre-mRNA splicing fail to gastrulate normally, display global defects in cell differentiation and proliferation and underexpress a number of genes including that encoding the conventional protein kinase C SpPKC1 [16].

Here we provide evidence that the gastrulation defect and abnormal proliferation associated with SpRunt-1 deficiency are secondary effects of extensive apoptosis that occurs in the post-blastula stage embryo. We further demonstrate that apoptosis is caused by a deficit in SpPKC1 activity, and that cell survival in SpRunt-1 morphant embryos can be rescued by exogenous *SpPKC1 *mRNA. Finally, we show that the *SpPKC1 *promoter region contains SpRunt-1 target sequences that are required for its transcriptional activation.

## Results and Discussion

### SpRunt-1 deficiency leads to apoptosis and secondary proliferation

**Figure 1:**
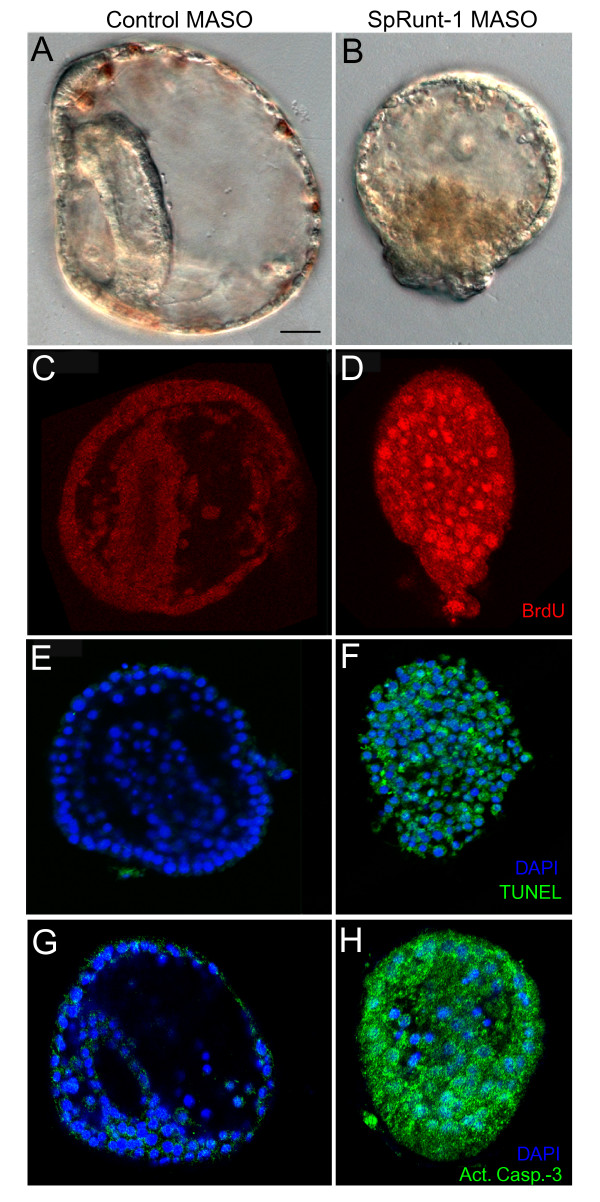
**Morpholino antisense-mediated knockdown of SpRunt-1 leads to ectopic proliferation and apoptosis in the gastrula stage (48 h) embryo**. (A-B) Differential interference contrast (DIC) images of embryos injected with (A) control MASO and (B) *SpRunt-1 *MASO. (C-D) Confocal micrographs (single optical sections) of BrdU-labeled embryos injected with (C) control MASO and (D) *SpRunt-1 *MASO. (E-F) Confocal sections of TUNEL (green)- and DAPI (blue, DNA stain)-labeled embryos injected with (E) control MASO and (F) *SpRunt-1 *MASO. (G-H) Confocal sections of antiactivated caspase-3 (green)- and DAPI (blue)-labeled embryos injected with (G) control MASO and (H) *SpRunt-1 *MASO. Scale bar = 20 μm.

To characterize further the abnormal cell proliferation associated with SpRunt-1 deficiency [16], gastrula stage embryos injected with either a nonspecific control MASO or the splice-blocking *SpRunt-1 *MASO were pulse-labelled with bromodeoxyuridine (BrdU). The embryos were fixed, and an anti-BrdU antibody was used to detect BrdU incorporation, which is indicative of DNA synthesis during the labelling period. Normal late gastrula stage embryos (Figure 1A) contained very few cells in S phase, as shown by an absence of nuclear BrdU signal in the controls (Figure 1C). In contrast, *SpRunt-1 *morphant embryos of the same age (Figure 1B) displayed extensive nuclear BrdU incorporation throughout the embryo (Figure 1D; 11/14 imaged embryos with phenotypes similar to that shown), indicating an abnormally high number of proliferating cells, in agreement with our previous cytometric DNA analysis of cell cycle distribution in these embryos [16].

**Figure 2:**
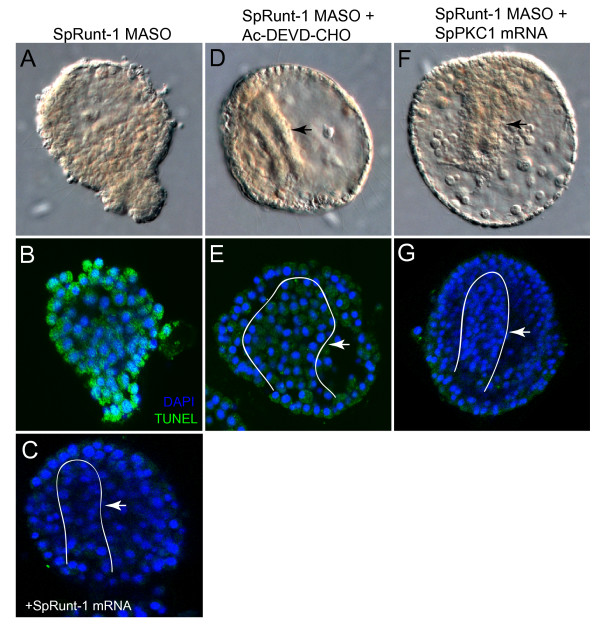
**Cell survival in *SpRunt-1 *morphant embryos is rescued by either injection of full-length *SpRunt-1 *mRNA, treatment with a caspase-3 inhibitor or injection of exogenous *SpPKC1 *mRNA**. (A) DIC image of 48 h *SpRunt-1 *morphant embryo. (B) Confocal section of 48 h *SpRunt-1 *morphant embryo labeled with DAPI (blue) and TUNEL (green). (C) Confocal section of 48 h *SpRunt-1 *morphant embryo that was coinjected with full-length *SpRunt-1 *mRNA and then labeled with DAPI and TUNEL as in (B). All of the imaged embryos (6/6) from this group had the TUNEL-negative phenotype shown, and 12/12 imaged embryos in a separate rescue experiment were similarly TUNEL-negative. (D) DIC image of 48 h *SpRunt-1 *morphant embryo, from same set of injected embryos as that shown in (A) but treated with the caspase-3 inhibitor Ac-DEVD-CHO. (E) Confocal section of 48 h *SpRunt-1 *morphant embryo treated with Ac-DEVD-CHO and then labeled with DAPI and TUNEL as in (B). In a separate experiment, the frequency of imaged Ac-DEVD-CHO-treated morphants displaying a TUNEL-negative phenotype similar to that shown here was 11/21, similar to the frequency of morphants displaying rescued gastrulation (Table 1). (F) DIC image of 48 h embryo injected with *SpRunt-1 *MASO + *SpPKC1 *mRNA. (G) Confocal section of 48 h embryo injected with *SpRunt-1 *MASO + *SpPKC1 *mRNA and then labeled with DAPI and TUNEL as in (B). Arrows point to invaginated guts, which are outlined in (C), (E) and (G).

Paradoxically, *SpRunt-1 *morphant embryos had about half as much DNA per embryo as gastrula stage controls of the same age [16]. Since deregulated cell proliferation is often associated with programmed cell death [17-19], we asked whether cells in *SpRunt-1 *morphants undergo apoptosis, which could account for these embryos displaying lower than normal DNA content despite having ectopic cell cycles. Terminal transferase-mediated dUTP nick end labeling (TUNEL) showed that, in contrast to controls (Fig. 1E), *SpRunt-1 *morphants displayed extensive DNA fragmentation (Figures 1F, 2B; 20/23 imaged embryos with phenotypes similar to that shown), a hallmark of apoptosis. Apoptosis was further indicated by the observation that *SpRunt-1 *morphants contained elevated levels of activated caspase-3 (Figure 1H; compare with control in Figure 1G) and caspase-6 (data not shown). The apoptosis was specifically caused by SpRunt-1 deficiency, as cell survival is rescued by coinjection of full-length *SpRunt-1 *mRNA with the splice-blocking *SpRunt-1 *MASO (Figure 2C). Interestingly, RNAi-mediated knockdown of the *Drosophila Runx *gene *CG15455 *was recently shown to lead to the apoptosis of in vitro cultured cells [20]; combined with our data this suggests an evolutionarily fundamental role for *Runx *genes in the control of metazoan cell survival.

Ectopic cell cycles in the *Drosophila *eye disc lead to compensatory apoptosis via Jun kinase (JNK) signaling [19]. We hypothesized that apoptosis in *SpRunt-1 *morphant embryos might similarly be a secondary response to unregulated cell proliferation, in which case inhibiting apoptosis might allow unbridled cell proliferation leading to a tumor-like overgrowth phenotype. To test this we used the caspase-3 inhibitor acetyl-Asp-Glu-Val-Asp-CHO (Ac-DEVD-CHO). Surprisingly, this drug rescued gastrulation (but not other aspects of development such as skeletogenesis) in about half of the *SpRunt-1 *morphants, allowing development of more normal looking embryos with fully invaginated guts (Figure 2D and Table 1). The rescued embryos displayed negligible apoptosis when assayed by TUNEL (Figure 2E), confirming the drug's efficacy in inhibiting caspase activity and suggesting that apoptosis is a principal cause of the gastrulation defect associated with SpRunt-1 deficiency. Apoptosis is also likely to be the underlying cause of the ectopic proliferation observed in these embryos (rather than the converse), as *SpRunt-1 *morphants rescued with the caspase-3 inhibitor displayed negligible BrdU incorporation at late gastrula stage, whereas *SpRunt-1 *morphants treated with the DNA polymerase inhibitor aphidicolin (which blocks cell proliferation while allowing for normal differentiation and morphogenesis from the mesenchyme blastula stage on [21]) were not rescued from apoptosis [see Additional file 1]. It is likely that the ectopic cell cycles associated with SpRunt-1 deficiency are a regulative response to a loss of embryo integrity, as occurs when cells from dissociated gastrula stage embryos re-enter the cell cycle [22].

### Apoptosis associated with SpRunt-1 deficiency is caused by loss of PKC expression

**Figure 3:**
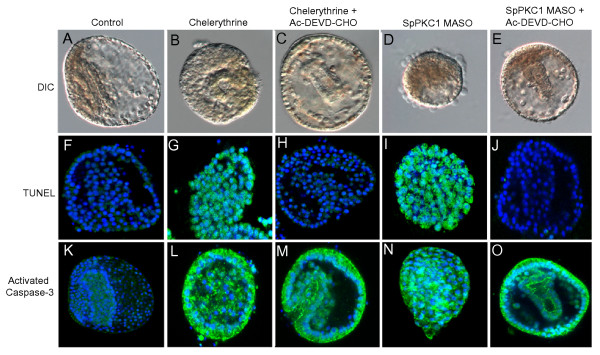
**Inhibition or knockdown of PKC leads to apoptosis that is suppressed by inhibition of caspase-3**. (A-E) DIC images, (F-J) fluorescent confocal images of embryos subjected to TUNEL (green) and counterstained with DAPI (blue) and (K-O) immunofluorescent confocal images of embryos stained for activated caspase-3 (green) and counterstained with DAPI (blue). (A), (F) and (K) are control embryos; (B), (G) and (L) are embryos treated with the PKC inhibitor chelerythrine from mesenchyme blastula stage; (C), (H) and (M) are embryos treated with chelerythrine and the caspase-3 inhibitor Ac-DEVD-CHO; (D), (I) and (N) are embryos injected with a *SpPKC1 *MASO; (E), (J) and (O) are embryos injected with the *SpPKC1 *MASO and treated with Ac-DEVD-CHO. The high levels of activated caspase-3 staining in panels (M) and (O) suggest that caspase-3 has been processed as a result of initiation of the apoptotic program but remains proteolytically inactive because of the presence of the inhibitor.

Protein kinase C (PKC) is a key component of numerous developmental signalling pathways, and the conventional PKC *SpPKC1 *was previously shown to be one of a number of genes that are significantly underexpressed in *SpRunt-1 *morphant embryos [16]. Moreover, mammalian PKCα promotes cell survival [23]. To ask whether the apoptosis associated with SpRunt-1 deficiency might be caused by the loss of PKC expression, we made use of chelerythrine, a compound that selectively and completely inhibits mammalian PKC activity at a concentration of ~10 μM [24]. Embryos treated with 2–4 μM chelerythrine from the mesenchyme blastula stage on (see Methods) displayed gastrulation defects (Figure 3B) as well as extensive apoptosis as indicated by TUNEL (Figure 3G) and elevated levels of activated caspase-3 (Figure 3L). A similar gastrulation-defective phenotype (Figure 3D; 80/101 embryos) and cell survival defect (Figure 3I, N) was obtained by injecting embryos with a MASO that targeted the translation initiation codon of *SpPKC1 *mRNA. In both cases cell survival (but not fully normal development) was rescued by treatment with the caspase-3 inhibitor (Figure 3C, H, E and 3J) despite the continued presence of active caspase-3 (Figure 3M, O; note that the inhibitor blocks the downstream activity of the activated caspase).

These data strongly suggest that the deficit in SpPKC1 expression in *SpRunt-1 *morphants might be the underlying cause of their apoptotic phenotype, in which case restoring *SpPKC1 *mRNA in these embryos should rescue cell survival. To test this, we coinjected zygotes with the *SpRunt-1 *MASO along with synthetic capped mRNA encoding SpPKC1. Unlike embryos injected with the *SpRunt-1 *MASO alone, most of the morphants coinjected with solutions containing 400–800 ng/μl of *SpPKC1 *mRNA gastrulated (Figure 2F and Table 2) and displayed negligible TUNEL signal (Figure 2G), indicating that underexpression of PKC is indeed the principal underlying cause of the apoptosis and resulting gastrulation defects associated with SpRunt-1 deficiency.

### SpRunt-1 target sequences in the *SpPKC1 *promoter region are required for transcriptional activation

**Figure 4:**
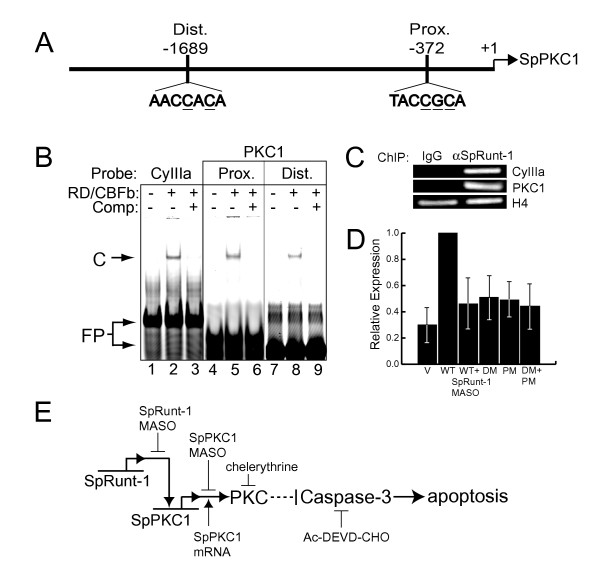
***SpPKC1 *is a direct target of SpRunt-1**. (A) Schematic of the 5' flanking sequence of *SpPKC1*, showing locations and sequences of the Runx consensus binding elements (underlined bases mutated for reporter gene studies). (B) EMSA of recombinant Runt domain (RD)-CBFβ heterodimers using probes consisting of target sequences from *CyIIIa *[12] and the distal and proximal Runx sites from *SpPKC1*. A 100-fold excess of unlabeled *CyIIIa *sequence was used as a competitor to test the specificity of the interactions. "C", complex between Runt domain and probe; "FP", free probe. (C) Chromatin immunoprecipitation (ChIP) from 48 h embryos using either an anti-*SpRunt-1 *[12] or nonimmune IgG. *CyIIIa *serves as a positive control, whereas *histone H4 *sequences (present as several hundred copies per haploid genome) serves as a negative control for specificity. (D) Relative normalized expression of wild-type (WT) *PKC1-luc *and mutant *PKC1-luc*, in which the proximal and distal Runx target sites have been mutated individually or in combination to abolish SpRunt-1 binding (PM, DM, and PM+DM, respectively), expression in control embryos and embryos injected with *SpRunt-1 *MASO. Fold difference in expression levels of each construct and of the basal luciferase vector alone (V) compared to the WT construct was measured by quantitative RT-PCR [38]. Each bar represents the averages and standard deviations from three to five different experiments. (E) Pathway summarizing the experimental approaches and results showing that SpRunt-1 promotes cell survival via transcriptional activation of *SpPKC1*.

Our findings raised the question of whether the requirement for SpRunt-1 in support of PKC expression is direct or indirect. Examination of the 5' flanking sequence of the *SpPKC1 *gene revealed the presence of two sequences that match the Runx target consensus within 1.7 kb of the first exon (Figure 4A and [see Additional file 2]). Fluorescently-labeled oligonucleotide probes representing each of these sites bound sequence-specifically to recombinant SpRunt-1 in an electrophoretic mobility shift assay (EMSA; Figure 4B). Furthermore, sequences from the *SpPKC1 *promoter region were specifically recovered by chromatin immunoprecipitation (ChIP) using an anti-SpRunt-1 antibody [12], indicating that SpRunt-1 is bound in vivo to these sequences at gastrula stage (Figure 4C). To test the functionality of the Runx target sequences in this region we constructed a *luciferase *reporter gene linked to ~1.9 kb 5' flanking sequence (including the endogenous promoter) of *SpPKC1 *(Figure 4A and [see Additional file 2]). When introduced into embryos, this sequence activated transcription of the reporter by approximately threefold and in a SpRunt-1 dependent fashion, as reporter expression was reduced to near baseline levels in *SpRunt-1 *morphant embryos (Figure 4D). Moreover, base substitutions that eliminate either the proximal or the distal Runx target sequences in the *SpPKC1 *cis-regulatory domain similarly reduced activity of the reporter, as did combined substitutions that eliminated both target sequences (Figure 4D), suggesting that the two Runx sites function synergistically rather than additively to activate transcription.

The relatively weak activation of the reporter conferred by the 1.9 kb *SpPKC1 *promoter fragment, together with our previous results showing that endogenous *SpPKC1 *expression is reduced approximately seven fold in *SpRunt-1 *morphant embryos [16], suggests that there may be additional enhancers outside of the 1.9 kb fragment that collaborate with SpRunt-1 in activating transcription of the endogenous gene. Testing this possibility would require cis-regulatory analysis using a larger genomic fragment that contains the *SpPKC1 *locus. Nevertheless, the data presented here support the proposition that the direct interaction of SpRunt-1 with its target sequences in the *SpPKC1 *cis-regulatory domain is required for normal levels of *SpPKC1 *transcription in the post-blastula stage embryo, which in turn is required to maintain PKC activity at levels sufficient to antagonize caspase activity and suppress apoptosis (Figure 4E).

## Conclusions

Our results support the idea that, in animal development, cells do not survive by default but rather by actively suppressing apoptosis in response to permissive cell signalling [25,26]. Furthermore, we find that deregulated apoptosis can lead to secondary cell proliferation, similar to the compensatory cell proliferation described by others [27,28]. In contrast to the latter phenomenon however, we do not observe overproliferation caused by apoptotic cell signalling in the caspase-3 inhibited embryos. While the specific role of SpPKC1 in suppressing apoptosis is not known, there are several plausible pathways based on well-known functions of mammalian PKCα, most notably its role in phosphorylating and thereby activating the anti-apoptotic protein Bcl-2. [29-31]. Identification of the relevant substrate(s) and pathway(s) in sea urchins awaits further analysis, as does the basis for the secondary proliferation observed in SpRunt-1 morphants.

In addition to the conventional PKCs (cPKCs), the PKC family also includes non-conventional and atypical PKCs (nPKCs and aPKCs, respectively) [32]. We do not yet know how many PKC family members are in *S. purpuratus*, although available expressed sequence tag (EST) and genomic sequence data suggest that *SpPKC1 *is the only *cPKC *gene expressed in the embryo, and possibly the only *cPKC *gene encoded in the genome (JAC, unpublished results). We note in closing that mammals have three *cPKC *genes, *PKC*α, *PKC*β, and *PKC*γ. PKCβ is actually pro-apoptotic in myeloid cells, and its gene was recently shown to be directly activated by a human Runx homolog, RUNX1 [33]. This suggests that Runx-mediated control of *cPKC *gene expression may be a primitive conserved character (i.e., plesiotypic) in animals, whereas the functional output of this interaction (cell death or survival) varies with context, something that would bear on whether a given *Runx *gene is an oncogene or tumor suppressor. It will therefore be interesting to determine whether the other mammalian *cPKC *genes are also direct Runx regulatory targets, and whether the Runx-dependency of cell survival in *Drosophila *[20] is a function of PKC.

## Methods

### Animals and gametes

Adult *Strongylocentrotus purpuratus *were purchased from either Charles Hollahan (Santa Barbara, California, USA) or Pat Leahy (Corona del Mar, California, USA). Gametes were obtained by vigorous shaking of animals or by intracoelomic injection of 0.5 M KCl.

### Morpholino antisense oligonucleotides

Morpholino antisense oligonucleotides (MASOs) were purchased from GeneTools, LLC (Philomath, Oregon, USA). Nonspecific control and splice-blocking *SpRunt-1 *(m5) MASOs were as described previously [16]. The sequence of the *SpPKC1 *MASO, which targets the AUG start codon of *SpPKC1 *mRNA, is: CATCTCAAACGACGAATCCGACATT.

### Microinjection and embryo culture

Microinjection of zygotes was carried out by the method of McMahon et al. [34], essentially as described in Kirchhamer et al. [35]. MASO injection solutions contained 500 μM MASO and 120 mM KCl. Embryos were cultured in artificial seawater at 15°C.

### BrdU labeling and antibody staining

Late gastrula stage embryos (44 h) were incubated in 300 μg/ml BrdU (Sigma-Aldrich; St. Louis, Missouri, USA) for four hours in artificial seawater (ASW) at 15°C then fixed in 4% formaldehyde in ASW for 90 min on ice. After the embryos were washed three times in ASW, they were treated with 2 M HCl on ice for 30 min, neutralized in 100 mM Tris, pH 9.5, 100 mM NaCl for 2 min on ice, and washed three times in phosphate buffered saline containing 0.2% Tween-20 (PBST). The embryos were post-fixed in 4% formaldehyde in PBST for 20 min on ice, and washed three times in ice-cold PBST. BrdU was detected by staining with an AlexaFluor 647-conjugated anti-BrdU antibody (Molecular Probes, Eugene, Oregon, USA) diluted 1:20 in PBST and incubated overnight at 4°C. Digital images were collected using a Leica TCS SP2 confocal microscope.

Antibodies directed against activated caspases 3 and 6 were obtained from Calbiochem (San Diego, California, USA). Embryos were stained with 5 μg/ml antibody in PBST with 5% bovine serum albumin (BSA) overnight at 4°C and then washed three times in PBST. Antibody labeling was detected by staining with AlexaFluor 488 goat anti-mouse IgG from Molecular Probes in PBST with 5% BSA for 1 h at room temperature. Following three washes in PBST and nucleic acid staining with 4',6-diamidino-2-phenylindole (DAPI), the digital images were obtained with the Leica TCS SP2 confocal microscope.

### Terminal transferase-mediated dUTP nick end labeling (TUNEL) assay

Late gastrula stage embryos (48 h) were fixed for 1 h in 4% paraformaldehyde at 4°C and washed for 1 h in PBST. Staining was performed using the In Situ Fluorescein Detection of Cell Death Kit from Roche (Indianapolis, Indiana, USA). Stained embryos were washed three times in PBST and labeled once in DAPI. Digital images were collected using a Leica TCS SP2 confocal microscope.

### DNA quantitation by fluorometry

The DNA content of isolated nuclei was quantitated by fluorometry as described previously [16].

### Drug treatments

The caspase-3 selective inhibitor Ac-DEVD-CHO (BD Biosciences; San Jose, California, USA) was added to *SpRunt-1 *morphant cultures at a final concentration of 50 μM at the 1–2 cell stage.

The DNA polymerase inhibitor aphidicolin (Sigma-Aldrich) was added to *SpRunt-1 *morphant cultures at a final concentration of 0.2–0.5 μg/ml at mesenchyme blastula stage (24 h post-fertilization).

The PKC inhibitor chelerythrine (Sigma-Aldrich) was added to embryo cultures at concentrations ranging from 2–4 μM from the mesenchyme blastula stage on. Higher concentrations tended to be embryonic lethal whereas lower ones gave no phenotype at all. Since the efficacy of the drug in producing the gastrulation-defective phenotype varied from culture to culture, the optimal concentration for producing the gastrulation-defective phenotype at a frequency close to 100% was determined empirically for each experiment. For example, in one experiment, 85% of embryos treated with 3.5 μM chelerythrine were viable but gastrulation defective (the remaining 15% appearing normal) whereas 100% of embryos treated with 4 μM chelerythrine were viable but gastrulation defective. For other cultures the effective dose for a similar effect was somewhat lower.

### Synthetic mRNAs

Full-length *SpRunt-1 *mRNA for rescue experiments was synthesized as previously described [16]. *SpPKC1 *mRNA for rescue experiments was obtained as follows: an EST (National Center of Biotechnology Information (NCBI) accession number CD290393) containing the *SpPKC1 *start codon was obtained by BLAST using the Genbank entry of *Lytechinus pictus PKC1 *(Genbank:U02967; [36]) as a query sequence. The *SpPKC1 *coding sequence was then assembled from the *S. purpuratus *genome traces using the *LpPKC1 *sequence as a scaffold. The assembled *SpPKC1 *sequence was used to design the following primers: GTGGATAATTTTTGCTGGATTTGCGCCTTGA, (forward; ends 13 bp upstream of start codon) and GTTCTACAGACAGACTGGCACTAGCT (reverse; reverse complement of stop codon underlined). These primers were used in a reverse transcription (RT)- PCR reaction using the Platinum Quantitative RT-PCR ThermoScript One-Step System from Invitrogen (Carlsbad, California, USA) and 200 ng total RNA (purified using the RNeasy Mini Kit from Qiagen; Valencia, California, USA) from 60 h embryos. An amplicon of the expected size was gel purified and inserted to pGEM-T Easy (Promega; Madison, Wisconsin, USA). Sequencing was performed to determine the insert orientation and also to confirm the identity of the *PKC1 *sequence. An Apa1-Spe1 restriction fragment containing the full length *SpPKC1 *sequence with some flanking sequence was then transferred into a pBluescript (Stratagene; LaJolla, California, USA) expression construct containing the *Xenopus *BetaGlobin 3' UTR and polyA sequence (pGloBlu) [16]. Pst1 was used to linearize the resulting construct for in vitro transcription of *SpPKC1 *using the T7 mMessage Machine from Ambion (Austin, Texas, USA).

### Assembly of the *SpPKC1 *5' flanking sequence

A contiguous sequence of over 2,500 bases extending in the upstream direction relative to the start codon of *SpPKC1 *was assembled from trace sequences obtained from the Sea Urchin Genome Project [11] using the ContigExpress program in the InforMax Vector NTI suite (Invitrogen). Genotrace [37], which supports the reassembly of short spans of genomic DNA sequence from trace files, was used to generate independently a contig for comparison with the manually assembled one in ContigExpress. The sequence was verified by PCR amplification from genomic DNA (see below).

### Electrophoretic mobility shift assay

Cy5 5'-modified oligonucleotides containing the Runx target sequences from the *SpPKC1 *gene were used as probes (see Additional file 2). The binding reaction contained 25 mM HEPES, pH 7.5, 75 mM NaCl, 10 mM MgCl_2_, 0.5 mM EDTA, 1 mM DTT, 0.3 mg/ml BSA, 10 μg/ml poly dIdC/dIdC and 8% glycerol. Approximately 10 nmol of purified recombinant protein was incubated for 15 min at room temperature with or without competitor and then incubated an additional 15 min at room temperature upon addition of the probe. Specificity of binding was determined by the addition of 100-fold molar excess of unlabeled competitor DNA corresponding to Runx target sequence from *CyIIIa *[12]. DNA-protein complexes were resolved on a 5% (w/v) nondenaturing polyacrylamide gel using 0.5× TBE running buffer at 150 V for 3 h at room temperature. Gels were imaged on a Typhoon 8700 (APBiotech; Piscataway, New Jersey, USA).

### Chromatin immunoprecipitation (ChIP)

Late gastrula stage embryos were dissociated into single cells by a washing once in dissociation buffer (1 M glycine pH 8, 2 mM EDTA) and then three times in calcium-free ASW. The dissociated cells were resuspended in a hypotonic buffer (10 mM HEPES, pH 7.9, 1.5 mM MgCl_2_, 10 mM KCl and protease inhibitors) and incubated for 15 min on ice. Igepal (10% solution) was then added to a final concentration of 0.6%, and the suspension was vortexed to release nuclei. Nuclei were recovered by centrifugation and resuspended in 20 mM HEPES, pH 7.9, 40 mM KCl, 0.1 mM EDTA, 1 mM DTT, 20% glycerol and protease inhibitor cocktail (Roche). Formaldehyde was then added to a concentration of 1%, and the nuclear suspension was incubated for 10 min at room temperature to crosslink the chromatin. After stopping the crosslinking reaction with the addition of glycine to 125 mM, the nuclei were recovered by centrifugation, resuspended in a nuclear lysis buffer (50 mM Tris-HCl, pH 8.1, 10 mM EDTA, 1% SDS and protease inhibitors) and sonicated with a force empirically determined to produce chromatin with an average fragment size of ~500 base pairs. The sonicated chromatin solution was then precleared with *Staphylococcus aureaus *protein A positive cells (Staph A) and aliquoted.

Prepared aliquots of chromatin were diluted with immunoprecipitation (IP) buffer (16.7 mM Tris-HCl, pH 8.1, 167 mM NaCl, 1.2 mM EDTA, 1.1% Triton X-100, 0.01% SDS and protease inhibitors) and incubated with equivalent amounts of either nonimmune or anti-SpRunt-1 IgG overnight at 4°C. The antibody complexes were recovered by precipitation with Staph A, and, following several washes of the precipitated Staph A with IP wash buffer (100 mM Tris-HCl, pH 9, 500 mM LiCl, 1% NP-40, 1% deoxycholic acid, and protease inhibitors), eluted with an SDS elution buffer (50 mM NaHCO_3_, 1% SDS). Following RNAase A addition (to 33 μg/ml), crosslink reversal by heat treatment (67°C for 5 h), ethanol precipitation and proteinase K treatment (240 μg/ml for 2 h at 45°C), the DNA from the immunoprecipitates was purified using the QIAquick PCR purification Kit (Qiagen), and used as template in PCR reactions with the following primers: *CyIIIa*: GTAGCACACGGAGAGATTGTGGGACAT (forward) and GGATCGGGGTTAGAGTTACATTTGGCTT (reverse); *SpPKC1*: CGAAACACACCACCAGCAACTTGCA (forward) and CCAATGGTCTTCAACACACACTTCGCTA (reverse); and *H4*: GTCGAGGAAAAGGAGGAAAGGGACTCGGAA (forward) and TGCTCGCAGTAGGTGACTGCATCACGGAT (reverse).

### SpPKC1:luciferase reporter gene construction and analysis

A 2.1 kb fragment of the upstream region of *SpPKC1 *was PCR amplified from genomic DNA, subcloned into pGEM-TEasy (Promega), and sequenced (see Additional file 2). The fragment was subsequently inserted into the pGLII Luciferase reporter plasmid (Promega) to generate the SpPKC1-Luc reporter. The primers used for PCR, GGGGTACC TGCACCCAATCCACACCCACTAAGA (forward) and CGGG ATCCACTGTCAAGGCGCAAATCCAGCAA (reverse), contain Kpn1 and BamH1 sites, respectively, which are compatible with the multiple cloning sequence in pGLII. The Quikchange Site Directed Mutagenesis Kit (Stratagene) was used to mutate each of the SpRunt-1 target sites within the SpPKC1-Luc reporter, using primers with the following sequences: ATCTCACCATAACgAtATAATTAGGGTTAGGAA (distal site forward), TTCCTAACCCTAATTATaTcGTTATGGTGAGAT (distal site reverse), TCATTCCTTTACATTTACgttAGTAGAAGTCAA (proximal site forward) and TTGACTTCTACTaacGTAAATGTAAAGGAATGA (proximal site reverse). The base substitutions introduced by these primers (shown in lower case) are known to abolish Runx binding [12] and were verified by sequencing of the resulting clones. Expression of *SpPKC1-Luc *and each *Runx*-site mutant was measured by quantitative real time RT-PCR essentially as described previously [16], and normalized to *luciferase *DNA content using the method of Revilla-i-Domingo et al [38]. The primers used to detect *luciferase *mRNA were GGGATTTCAGTCGATGTACACGTTCGTCACATCT (forward) and GGCATGCGAGAATCTGACGCAGGCAGTTCTAT (reverse).

## Authors' contributions

CED performed the microinjections, embryo labeling, imaging and counts of embryos, and measurements of *luciferase *expression. AJR made the plasmid constructs, assembled the *SpPKC1 *promoter and obtained sequence from a cloned PCR amplicon from sea urchin genome data and assisted with ChIP. DER performed ChIP and EMSA with assistance from JJM, who developed the ChIP protocol. JAC directed the research, assisted with some of the microinjections, assisted with microscopy and data analysis and drafted the manuscript and figures.

## Supplementary Material

Additional File 1 **Supplemental Figure 1 – In *SpRunt-1 *morphant embryos, the caspase-3 inhibitor Ac-DEVD-CHO suppresses ectopic cell proliferation, whereas the DNA polymerase inhibitor aphidicolon does not suppress the apoptosis**. (A-C) Gastrula stage embryos labeled with BrdU and DAPI. (D-F) Gastrula stage embryos labeled with TUNEL and DAPI. (A and D) Control MASO injected embryos. (B, C, E and F) *SpRunt-1 *MASO injected embryos. (C) Embryo treated with Ac-DEVD-CHO. (F) Embryo treated with aphidicolin from mesenchyme blastula stage on (as was the control embryo shown in D). Click here for file

Additional File 2 **Supplemental Figure 2 – 5' flanking sequence from the *SpPKC1 *gene**. The sequence was assembled from overlapping trace sequences obtained from the sea urchin genome [11], a sequenced clone derived from a PCR amplicon (in boldface; primer sites at termini predicted from assembled genomic traces) and an EST in the NCBI database (solid underlined sequence) that matches the N terminus of *LpPKC1 *coding sequence (96% identity, 142 residues). Dotted underlined sequence corresponds to the 5' UTR of the full length *LpPKC1 *cDNA (which begins with the sequence: ACGAACATTT). Runx target sequences used to make probes for EMSA are highlighted in color (core in yellow, flanking sequences in green); lower case letters indicate residues that vary between the sequenced clone (shown) and the trace genome sequences. Coding sequence is highlighted in grey. A putative non-canonical TATA box located 25 bases upstream of the inferred transcriptional start site is highlighted in black. Click here for file

## Figures and Tables

**Table 1:** Rescue of gastrulation in *SpRunt-1 *morphants by Ac-DEVD-CHO. Embryos injected with *SpRunt-1 *MASO were divided into two groups, one of which was treated with Ac-DEVD-CHO as described in the Methods. At 48 h the embryos were observed under a stereomicroscope and scored as gastrulation defective (with a typical morphant phenotype similar to that depicted in Figure 2A), rescued gastrulation (with guts but no skeletons, a phenotype similar to that depicted in Figure 2D), or normal (indistinguishable from uninjected control embryos with skeletons). The latter group probably represents embryos that were under injected; note that this particular experiment had an unusually high proportion of such embryos (which more typically are in the range of 10–15%).

Treatment	Gastrulation defective	Rescued Gastrulation	Normal
*SpRunt-1 *MASO alone (n = 259)	162 (63%)	-	97 (37%)
*SpRunt-1 *MASO + Ac-DEVD-CHO (n = 158)	59 (37%)	55 (35%)	44 (28%)

**Table 2:** Rescue of gastrulation in SpRunt-1 knockdown embryos by *SpPKC1 *mRNA. Zygotes were coinjected with *SpRunt-1 *MASO and varying amounts of *SpPKC1 *mRNA in two different experiments. At 48 h embryos were observed under a stereomicroscope and scored for successful gastrulation (fully invaginated guts, with a phenotype similar to that depicted in Figure 2F). The *SpPKC1 *[mRNA] refers to the concentration of the injection solution, approximately 2–5 pl of which was injected into each zygote.

Experiment	*SpPKC1 *[mRNA] (ng/μl)	Successful Gastrulation
1	200	2/50 (4%)
	400	13/50 (26%)
	800	36/50 (72%)
		
2	0	15/120 (12.5%)
	200	48/100 (48%)
	400	138/155 (89%)

## References

[B1] Coffman JA (2003). Runx transcription factors and the developmental balance between cell proliferation and differentiation. Cell Biol Int.

[B2] Ito Y (2004). Oncogenic potential of the RUNX gene family: 'overview'. Oncogene.

[B3] Lund AH, van Lohuizen M (2002). RUNX: a trilogy of cancer genes. Cancer Cell.

[B4] Cameron ER, Neil JC (2004). The Runx genes: lineage-specific oncogenes and tumor suppressors. Oncogene.

[B5] Bernardin-Fried F, Kummalue T, Leijen S, Collector MI, Ravid K, Friedman AD (2004). AML1/RUNX1 increases during G1 to S cell cycle progression independent of cytokine-dependent phosphorylation and induces cyclin D3 gene expression. J Biol Chem.

[B6] Bernardin F, Friedman AD (2002). AML1 stimulates G1 to S progression via its transactivation domain. Oncogene.

[B7] Strom DK, Nip J, Westendorf JJ, Linggi B, Lutterbach B, Downing JR, Lenny N, Hiebert SW (2000). Expression of the AML-1 oncogene shortens the G(1) phase of the cell cycle. J Biol Chem.

[B8] Linggi B, Muller-Tidow C, van de Locht L, Hu M, Nip J, Serve H, Berdel WE, van der Reijden B, Quelle DE, Rowley JD, Cleveland J, Jansen JH, Pandolfi PP, Hiebert SW (2002). The t(8;21) fusion protein, AML1 ETO, specifically represses the transcription of the p14(ARF) tumor suppressor in acute myeloid leukemia. Nat Med.

[B9] Lutterbach B, Westendorf JJ, Linggi B, Isaac S, Seto E, Hiebert SW (2000). A mechanism of repression by acute myeloid leukemia-1, the target of multiple chromosomal translocations in acute leukemia. J Biol Chem.

[B10] Westendorf JJ, Zaidi SK, Cascino JE, Kahler R, van Wijnen AJ, Lian JB, Yoshida M, Stein GS, Li X (2002). Runx2 (Cbfa1, AML-3) interacts with histone deacetylase 6 and represses the p21(CIP1/WAF1) promoter. Mol Cell Biol.

[B11] Sea Urchin Genome Project. http://www.hgsc.bcm.tmc.edu/projects/seaurchin/.

[B12] Coffman JA, Kirchhamer CV, Harrington MG, Davidson EH (1996). SpRunt-1, a new member of the runt domain family of transcription factors, is a positive regulator of the aboral ectoderm-specific CyIIIA gene in sea urchin embryos. Dev Biol.

[B13] Rennert J, Coffman JA, Mushegian AR, Robertson AJ (2003). The evolution of runx genes. I. A comparative study of sequences from phylogenetically diverse model organisms. BMC Evol Biol.

[B14] Robertson AJ, Dickey CE, McCarthy JM, Coffman JA (2002). The expression of SpRunt during sea urchin embryogenesis. Mech Dev.

[B15] Stricker S, Poustka AJ, Wiecha U, Stiege A, Hecht J, Panopoulou G, Vilcinskas A, Mundlos S, Seitz V (2003). A single amphioxus and sea urchin runt-gene suggests that runt-gene duplications occurred in early chordate evolution. Dev Comp Immunol.

[B16] Coffman JA, Dickey-Sims C, Haug JS, McCarthy JJ, Robertson AJ (2004). Evaluation of developmental phenotypes produced by morpholino antisense targeting of a sea urchin Runx gene. BMC Biology.

[B17] Fotedar R, Diederich L, Fotedar A (1996). Apoptosis and the cell cycle. Prog Cell Cycle Res.

[B18] Li QJ, Pazdera TM, Minden JS (1999). Drosophila embryonic pattern repair: how embryos respond to cyclin E-induced ectopic division. Development.

[B19] Brumby AM, Richardson HE (2003). scribble mutants cooperate with oncogenic Ras or Notch to cause neoplastic overgrowth in Drosophila. Embo J.

[B20] Boutros M, Kiger AA, Armknecht S, Kerr K, Hild M, Koch B, Haas SA, Consortium HF, Paro R, Perrimon N (2004). Genome-wide RNAi analysis of growth and viability in Drosophila cells. Science.

[B21] Stephens L, Hardin J, Keller R, Wilt F (1986). The effects of aphidicolin on morphogenesis and differentiation in the sea urchin embryo. Dev Biol.

[B22] De Petrocellis B, Filosa-Parisi S, Monroy A, Parisi E (1977). Cell interactions and DNA replication in the sea urchin embryo. Soc Gen Physiol Ser.

[B23] Whelan RD, Parker PJ (1998). Loss of protein kinase C function induces an apoptotic response. Oncogene.

[B24] Chmura SJ, Dolan ME, Cha A, Mauceri HJ, Kufe DW, Weichselbaum RR (2000). In vitro and in vivo activity of protein kinase C inhibitor chelerythrine chloride induces tumor cell toxicity and growth delay in vivo. Clin Cancer Res.

[B25] Raff MC (1992). Social controls on cell survival and cell death. Nature.

[B26] Kong M, Fox CJ, Mu J, Solt L, Xu A, Cinalli RM, Birnbaum MJ, Lindsten T, Thompson CB (2004). The PP2A-associated protein alpha4 is an essential inhibitor of apoptosis. Science.

[B27] Ryoo HD, Gorenc T, Steller H (2004). Apoptotic cells can induce compensatory cell proliferation through the JNK and the wingless signaling pathways. Dev Cell.

[B28] Huh JR, Guo M, Hay BA (2004). Compensatory proliferation induced by cell death in the Drosophila wing disc requires activity of the apical cell death caspase Dronc in a nonapoptotic role. Curr Biol.

[B29] Bai XC, Deng F, Liu AL, Zou ZP, Wang Y, Ke ZY, Ji QS, Luo SQ (2002). Phospholipase C-gamma1 is required for cell survival in oxidative stress by protein kinase C. Biochem J.

[B30] Ito T, Deng X, Carr B, May WS (1997). Bcl-2 phosphorylation required for anti-apoptosis function. J Biol Chem.

[B31] Ruvolo PP, Deng X, Carr BK, May WS (1998). A functional role for mitochondrial protein kinase Calpha in Bcl2 phosphorylation and suppression of apoptosis. J Biol Chem.

[B32] Parker PJ, Murray-Rust J (2004). PKC at a glance. J Cell Sci.

[B33] Hug BA, Ahmed N, Robbins JA, Lazar MA (2004). A chromatin immunoprecipitation screen reveals protein kinase Cbeta as a direct RUNX1 target gene. J Biol Chem.

[B34] McMahon AP, Flytzanis CN, Hough-Evans BR, Katula KS, Britten RJ, Davidson EH (1985). Introduction of cloned DNA into sea urchin egg cytoplasm: replication and persistence during embryogenesis. Dev Biol.

[B35] Kirchhamer CV, Davidson EH (1996). Spatial and temporal information processing in the sea urchin embryo: modular and intramodular organization of the CyIIIa gene cis-regulatory system. Development.

[B36] Rakow TL, Shen SS (1994). Molecular cloning and characterization of protein kinase C from the sea urchin Lytechinus pictus. Develop Growth Differ.

[B37] Genotrace. http://genotrace.niob.knaw.nl/.

[B38] Revilla-i-Domingo R, Minokawa T, Davidson EH (2004). R11: a cis-regulatory node of the sea urchin embryo gene network that controls early expression of SpDelta in micromeres. Dev Biol.

