# Supplementary figures and images for: Runx-dependent expression of PKC is critical for cell survival in the sea urchin embryo
Source: BMC Biol. 2005 Aug 2;3:18. doi: 10.1186/1741-7007-3-18 (PMC1187879; doi:10.1186/1741-7007-3-18)

Control MASO

SpRunt-1 MASO

A

B

C

Ac-DEVD-CHO

BrdU

D

E

F

Aphidicolin

TUNEL

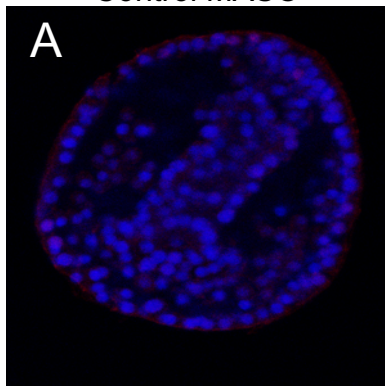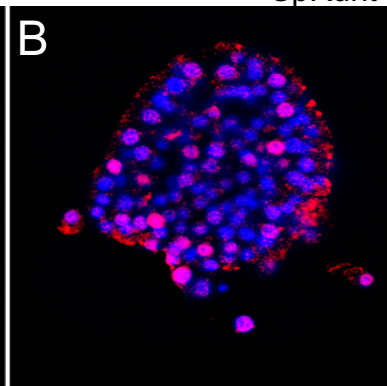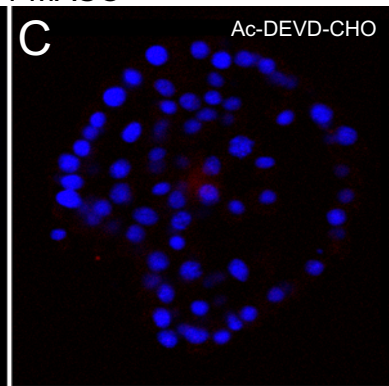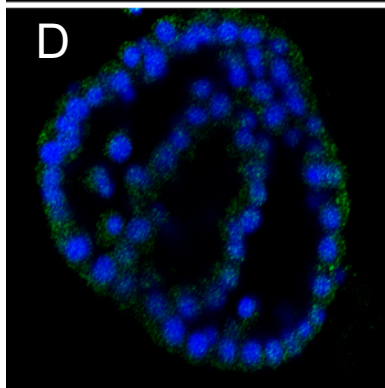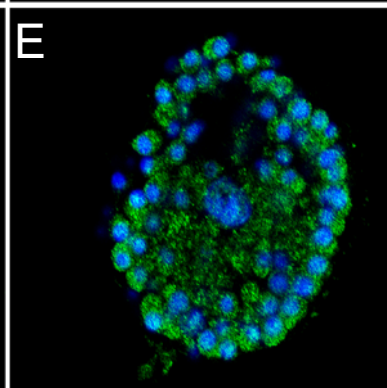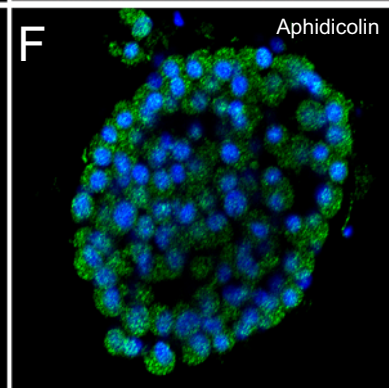

Supplement: Additional File 1 — Supplemental Figure 1 – In SpRunt-1 morphant embryos, the caspase-3 inhibitor Ac-DEVD-CHO suppresses ectopic cell proliferation, whereas the DNA polymerase inhibitor aphidicolon does not suppress the apoptosis. (A-C) Gastrula stage embryos labeled with BrdU and DAPI. (D-F) Gastrula stage embryos labeled with TUNEL and DAPI. (A and D) Control MASO injected embryos. (B, C, E and F) SpRunt-1 MASO injected embryos. (C) Embryo treated with Ac-DEVD-CHO. (F) Embryo treated with aphidicolin from mesenchyme blastula stage on (as was the control embryo shown in D). [file 1741-7007-3-18-S1.pdf]
