# Supplementary material for: Runx-dependent expression of PKC is critical for cell survival in the sea urchin embryo
Source: BMC Biol. 2005 Aug 2;3:18. doi: 10.1186/1741-7007-3-18 (PMC1187879; doi:10.1186/1741-7007-3-18)
Supplement: Additional File 2 — Supplemental Figure 2 – 5' flanking sequence from the SpPKC1 gene. The sequence was assembled from overlapping trace sequences obtained from the sea urchin genome [11], a sequenced clone derived from a PCR amplicon (in boldface; primer sites at termini predicted from assembled genomic traces) and an EST in the NCBI database (solid underlined sequence) that matches the N terminus of LpPKC1 coding sequence (96% identity, 142 residues). Dotted underlined sequence corresponds to the 5' UTR of the full length LpPKC1 cDNA (which begins with the sequence: ACGAACATTT). Runx target sequences used to make probes for EMSA are highlighted in color (core in yellow, flanking sequences in green); lower case letters indicate residues that vary between the sequenced clone (shown) and the trace genome sequences. Coding sequence is highlighted in grey. A putative non-canonical TATA box located 25 bases upstream of the inferred transcriptional start site is highlighted in black. [file 1741-7007-3-18-S2.doc]

GAGAAAGAGGAATAGAAAGCAATATCGACAGATCAAGAATTCTGGTAACTATGCCCGGAAAAGATCACGAGGCCATTCTAGAGAATCTGAAATAGATAGTGATAAGGCGGGAGAAATAAATTTAATATTACGCCGAAATAGGACAGAATCCCTTAACAAGAATAATACAAAGAAACTCCAATGATTATAAGGTAATATAGTATAGGGAACCTTTCAAAGACTAATATATAAATTAAGTCCCACATTAATAGATTTTATAAAATAAGAGAGAATAAAAAGAAATATTTAAAAGCGAAATAAGCTGGGTTACCCACGCCTCCAGAGTTAAAGGTCATAAGTATAAGGATAACCAAGTTCTACGCAGTAAACCGGAAGGGAAAGAGATTTAGGCCTAATATTACCAGATCCACCAATTCAGAATTTTGGGGAAGGGAAAATAACTGAAGCTGGCTAGGGTTGTGTT**TGCACCCAATCCACACCCACTAAGAAAATAAGGTGCAATAAACCCCTCCATAACCGCACCCTATAATGGGAGCAATTATGAGGGTGCAGACATTTTGCTACAAAAGGGTGCAATTCTTGTTTTGGGTGTTTTTTTTTTTTGGGGGAGGGGTGGGTGGTCTTTTTTGTCTTTTAGAATATCTCACCATAACCACAatATTAGGGTTAGGAATAGACCGAGGGATTAGATAAAAAGGAAATTAGAATGGTGGGGGTAATATCCTCCAATGCAGGGACAGGATTCCGGGAACAGAGATATTTTCTAAAAGTAAAAGTTTGGAATGAATTAAAATTCTGTCTCAGTATATGTTAATTCATGAAAAAGGAATCTCCAGCCTACCCCTGGCCTAATTTTTCATTTCTTATAAAATGTTAACAAAATAATTAACAAAACACGTGATTTTTCATCCCAATTTCAATAAAACCTAACCTGTACCCACTGCGGCCTTTTTATTTTTAATATTCAATACCAGTAAAGTTTTCTACATAAAATCTAATTCTATTTATATTGAATTCTAATTCATGCAAATTAAAAAAATTAGGCTCAGGCCCATAAAGGCCCTTTTGGCCAAATTTTCAATTTCTAAAAATACAAAAAAAATATATTTCCATCAAAATGCTAGGCACGATAAAAGTCGTGTTTTGGCCTGGCGTTTTGGACTATATGCGATTTTCTATACCAATTCAATAAAACCATACCCAGACCACGAAAATTTCCTCTGAAATAGAAAATGCTTTTAGTTTAAAGTCTCTGAAGTTCCGGACTGTTTCATCATCAATGTTGTATTTGTTCTTCTCAAAACCCACCTTTTTAATCAGCAGTATACTGGAGGCGCACACAAGTAGTTAAATCAATTTAACTGAATTGTATTAGTAATATTTCATTTCATTTTGTTGCTTTTAAGCTATTGAACATGTAAAATAATTATACTGAGTTAGACAGTACCACCTGTGTGCTTACTAACACCTGTATAAAGCGCCCTATAAATGTAGTTATTATTATTATTAATATTAGAATCAGTAACTGTTCATTGTTCTTGTGTCATTTACTTAATATAAAATAGATCAAATTTGAAATGTAATTCTTTTCTTCAGAGAATAAATACATGTTAACATTGTGAATTTTACATCAGAAGCGCAATTTGATTTATTTTTAAAATAAATTACTGCATTGTCAACAACTTCAATGGTAATCACTATCGCACTTCAAAATTCTCAAGGGGTTACAATTTTACAAAACTTACTTGTTTAGGAGTACACTTTTGTGAAATGCTTGTTTAGGGTGAGAATTCGGGAACATTTTTGGCTTCAGAATAAATCCTCGTTTAGGGGGGGGGGGGGGTGTTTGGGACACGCATGCTTACAGTATTTCTTCATGACCGAGACCCCTGGCTTAATATGTTGATGTGTTTGTTAAACTTGTGATACCATGGTGCACCTTCTTGGTTAAAGTAAGTtcaTtCcTTTACCGCAGTAGAAGTCAATATGTTATATTTGATGCAGATTAATATTGATATTTAAAGATTAGAAATACAAACAGATTCATTCAAGTGTTCTTTAAATACATTCACACTAGCGCTAGCGAGCTTTCTTTCTTCTTTCTTTCTTGATTTGATTTTTTGTTTGGCAATCAGTCACAGTTGACTATTTTTGCCAAATATTTTTGCACTCCGATCCAGTCTAGACTCTTCGCGAGCGGTTTCATTGGAATTATTAAAAATCGAGAAATTCGCTTCGCTAGCGCGCCCCACTGGCCGAAACACACCACCAGCAACTTGCATGTCAGCTAGCTATTGATACAGTGTGCAGTATACACTCAACGCGTTATTGGCGATCTCACGCACATTTTCCGTAGGTACGTGCTTTACATGTGCACGTACAGCCATACCACTGAGGCGCTAGGCTCCTAGTCATCGCTCGCTCTATTGGAGTCCACTTATTAGCGAAGTGTGTGTTGAAGACCATTGGATGTGCCGAAAAAATCGGTTAGGGGTAGAAGCATACTTTCATTTATAAAAAGTGGATAATTTTTGCTGGATTTGCGCCTTGACAGTGGAT**TTATAATGTCGGATTCTTCGTTTGAGATGAAGGGATTCGCTAGGAGGGGAGCGTTACGTCAGAAGAACGTGTATGAGATCAAAAATCACAAATTCATCCCGAGGTTTTTCAAGCAGCCGACATTTTGTAGTCATTGCAAGGATTTCATATGGTAAGTACACACGTACTGTACATGGCGGATGCACTGACCTCGCAAATACCAACGAATCAGGTCATATCTATTAAGTATTATAAATGATCATTCATTCATCACAGCGATCATGTATTTAGGCTTTGCGATTGAATTG
